# PROTOCOL: Psychosocial, pharmacological and legal interventions for improving the psychosocial outcomes of children with substance misusing parents

**DOI:** 10.1002/cl2.1113

**Published:** 2020-09-02

**Authors:** Elizabeth Eggins, Sharon Dawe, David B. Wilson, Ned Chandler‐Mather, Joseph Betts

**Affiliations:** ^1^ School of Applied Psychology, Griffith University Mount Gravatt Queensland Australia; ^2^ Department of Criminology George Mason University Fairfax Virginia USA

## Abstract

This review aims to first enhance and update existing reviews by comprehensively synthesising the full array of psychosocial, pharmacological and legal interventions that aim to improve the psychosocial outcomes of children with substance misusing parents. Second, the review aims to use network meta‐analysis to integrate and examine the comparative impact of these interventions. Specifically, the review will address the following research questions: (1) What is the comparative impact of psychosocial, pharmacological, and legal interventions for improving the psychosocial outcomes of children with substance misusing parents? (2) Does the impact of interventions vary according to the child developmental period (e.g., infancy, early childhood, adolescence) or the type of (a) outcome measure; (b) substance misuse; (c) practitioner implementing the intervention; or (d) intervention setting? (3) Does the impact of interventions vary by the country of implementation?

## 1 BACKGROUND

### 1.1 The problem, condition or issue

An extensive body of literature documents the adverse outcomes of children who are raised in families with parental substance abuse. These include increased risk and reports of child abuse and neglect (Taplin, Saddichha, Li, & Krausz, 2014; Wekerle, Wall, Leung, & Trocme, 2007; Williams, Tonmyr, Jack, Fallon, & MacMillan, 2011), poor cognitive development and educational attainment (Lambert & Bauer, 2012; Park & Schepp, 2014; Richardson, Goldschmidt, Larkby, & Day, 2015), psychopathology (Bountress & Chassin, 2015; Christoffersen and Soothill, 2003; Hser, Evans, Li, Metchik‐Gaddis, & Messina, 2014; Marmorstein, Iacono, & McGue, 2009; Vidal et al., 2012), and adolescent substance misuse and antisocial behaviour (Burlew et al., 2013; Clark, Cornelius, Kirisci, & Tarter, 2005; King, Meehan, Trim, & Chassin, 2006; King et al., 2009; Lambert et al., 2013; Lambert & Bauer, 2012; Walden, Iacono, & McGue, 2007).

Parental substance misuse typically co‐occurs in the context of multiple risk factors across domains of parent and family functioning. These include parental psychopathology and criminality, domestic violence, and severe poverty (e.g., see Grella, Hser, & Huang, 2006; Hser et al., 2015; Miller, Orellana, Johnson, Krase, & Anderson‐Nathe, 2013; Skinner, Haggerty, Fleming, Catalano, & Gainey, 2010). Thus, the accumulation and interplay between risk factors, rather than parental substance abuse per se, results in multiple and complex family environments that place considerable challenges on parents, which then contributes to poor child outcomes (e.g., see Conners et al., 2004; Nair, Schuler, Blacka, Kettinger, & Harrington, 2003; Velleman & Templeton, 2007). Neger and Prinz (2015) propose a conceptual framework with multiple interrelated pathways to explain how parental substance misuse directly and indirectly impacts risk factors predictive of poor child outcomes (see also Dunn et al., 2002; Eiden, Coles, Schuetze, & Colder, 2014; Finger, Schuetze, & Eiden, 2014; Miller, Orellana, Briggs, & Quinn, 2014; Shorey et al., 2013; Twomey et al., 2013). For example, parents with substance misuse issues often have difficulty regulating negative emotional states or experience co‐occurrence of mental health disorders (Smithet al., 2012; Whitaker, Orzol, & Kahn, 2006), which can impact their capacity to assess and attend to their child's emotional well‐being and needs (Borelli, Luthar, & Suchman, 2010; Borelli, West, Decoste, & Suchman, 2012; Siqveland and Moe, 2014) or responsively parent their child according to child developmental needs (Slesnick, Feng, Brakenhoff, & Brigham, 2014; Velez et al., 2004). A history of trauma and childhood adversity is common in parents with substance misuse problems (Hatzis, Dawe, Harnett, & Loxton, 2019) and this, combined with the risk factors above, make sensitive and responsive caregiving challenging (Hatzis, Dawe, Harnett, & Barlow, 2017). Importantly, deficits in parent emotional regulation and the capacity to responsively parent are key predictors of child abuse and maltreatment (Stith et al., 2009).

Global estimates indicate that ∼5–10% of all children are being raised in families with one or more parent who misuses alcohol or other drugs (Dawe et al., 2006; Jääskeläinen, Holmila, Notkola, & Raitasalo, 2016; Manning, Best, Faulkner, & Titherington, 2009; Raninen, Elgán, Sundin, & Ramstedt, 2015; SAMHSA, 2014). This prevalence and the complexities and enduring challenges associated with parental substance misuse has led to the development of a range of approaches that aim to reduce risk factors, enhance family functioning, and improve child outcomes. Importantly, recent estimates suggest that for every dollar invested into substance misuse treatment, there are significant cost savings for society (Dalziel, Dawe, Harnett, & Segal, 2015; National Institute of Drug Abuse, 2012; Public Health England, 2014). However, a critical limitation of the current evaluation and review literature is the lack of integration and synthesis of the *relative* effectiveness of different intervention models that aim to improve the outcomes for children with substance misusing parents. Without a clear understanding of the relative effectiveness of different intervention approaches, practitioners and policy‐makers are limited in their ability to make informed and reliable choices between intervention models. Therefore, the proposed review aims to provide a comprehensive up‐to‐date review of psychosocial, pharmacological, and legal interventions in the context of parental substance misuse and the impact of these interventions on child psychosocial outcomes. Moreover, the review will provide a unique contribution by using network meta‐analysis (NMA) to synthesise the comparative effectiveness of these different intervention approaches (see Hutton et al., 2015; Mavridis, Giannatsi, Cipriani, & Salanti, 2015; Salanti, 2012; Wilson, Tanner‐Smith, & Mavridis, 2015).

### 1.2 The intervention

In order to conduct an NMA, this review will include all possible interventions that explicitly aim to improve the psychosocial well‐being of families in which at least one parent has either a current substance misuse problem or is in treatment for substance misuse problems. The focus of the review will be on studies that examine the impact of psychosocial, legal and/or pharmacological interventions on child psychosocial outcomes. For the purposes of this review, we draw on Maynard et al. (2015) to define a psychosocial intervention to encompass those that are implemented by professional practitioners (e.g., clinicians, social workers, teachers) across a variety of settings (e.g., homes, school, community, clinics, residential facilities and/or hospitals) that aim to address psychological and social well‐being more generally. We draw on Eggins et al. (2020) and Mazerolle, Eggins, and Sydes (2018) and define a legal intervention to be “some kind of a strategy, technique, approach, activity, campaign, training, directive, or funding or organisational change that involves the criminal justice system” (p. 21). We define pharmacological interventions to be medication or pharmacy‐related approaches to treating parental substance misuse (e.g., buprenorphine or morphine). Examples of eligible interventions are briefly described below (not exhaustive) and additional inclusion specifications are provided in Section 3.

#### 1.2.1 Home visiting

Home‐visiting interventions are characterised by regular home visits made by health practitioners, such as nurses, or paraprofessionals to either pregnant mothers or mothers and their young infants. Home‐visiting programmes generally aim to improve the psychosocial and health outcomes for mothers and infants (Segal, Sara Opie, & Dalziel, 2012; Turnbull & Osborn, 2012). This category of interventions generally begins in either the prenatal or early postpartum period and the duration of the intervention can span from weeks to years (Segal et al., 2012). The specific content of home‐visiting interventions varies and can include psychoeducation, health surveillance, connection with community resources, parent training and/or counselling components (Turnbull & Osborn, 2012). Home‐visiting interventions with substance misusing parents have been evaluated with randomised controlled trials using child psychosocial outcomes US and non‐US locations (e.g., Butz et al., 2001; Quinlivan, Box, & Evans, 2003; Schuler, Nair and Kettinger, 2003).

#### 1.2.2 Family treatment drug courts

Family treatment drug courts (FTDC) are specialised problem‐solving courts that use a nonadversarial and treatment‐oriented approach for managing cases where both parental substance misuse and child maltreatment have been identified as issues (Gifford, Eldred, Vernerey, & Sloan, 2014; Gifford, Sloan, Eldred, & Evans, 2015; Lloyd, 2015). Cases dealt within FTDCs have a dual focus on promoting the safety and well‐being of children and families and also the treatment of parents' substance misuse, whereby withdrawal or retention of parental rights is used as leverage for treatment compliance (Dakof et al., 2010; Gifford et al., 2014; 2015; Lloyd, 2015). Although the exact process and content can slightly differ across jurisdictions, common FTDC components include: (a) multidisciplinary teams of professionals who collaborate with families to devise a holistic case‐plan to address parental substance misuse and child welfare issues; (b) frequent court hearings and drug testing to monitor treatment adherence and case progress; (c) incentives or rewards for compliance; and (d) sanctions for noncompliance (Chuang, Moore, Barrett, & Young, 2012; Edwards & Ray, 2005; Haack, Alemi, Nemes, & Cohen, 2005). FTDCs have been most widely evaluated in the United States (e.g., Ashford, 2004; Worcel, Furrer, Green, Burrus, & Finigan, 2008), yet have recently emerged in the United Kingdom (Bambrough, Shaw, & Kershaw, 2014) and Australia (Marshall, 2015).

#### 1.2.3 Multidimensional interventions

In order to address the accumulation of risks across multiple ecological domains characteristic of families with parental substance misuse issues, a number intervention models integrate substance misuse treatment with other biopsychosocial treatments (Marsh, Smith, & Bruni, 2011; Niccols et al., 2012; Uziel‐Miller & Lyons, 2000). These multidimensional interventions aim to comprehensively treat parental substance misuse, alleviate other psychosocial risks and minimise barriers to treatment by simultaneously providing intervention components across different ecological domains (Niccols et al., 2012). Common components include: substance misuse treatment (pharmacological and/or psychological support around substance misuse), mental health services, flexible and accessible delivery (e.g., providing transportation and childcare or visiting homes), medical services for family members (e.g., prenatal care, immunisations for children), parenting programmes, vocational and education assistance and other support services (e.g., housing, financial or legal services). Multidimensional interventions have been evaluated or are currently registered for evaluation using randomised controlled or quasi‐experimental trials and child psychosocial outcomes measures in a number of countries (e.g., Barlow et al., 2013; Catalano, Gainey, Fleming, Haggerty, & Johnson, 1999; Dawe & Harnett, 2007; Field et al., 1998; Noether et al., 2007).

#### 1.2.4 Family, parent or child‐focused interventions

Interventions within this category can be distinguished from those in the above‐mentioned categories based on their narrower intervention focus. Generally, these interventions target the family unit, parents or children in the absence of more intensive case‐management components. For example, the “Strengthening Families” programme consists of concurrent parent training, child training to promote coping, communication and resistance skills and joint family sessions to facilitate the transfer of acquired knowledge and skills (see Renk et al., 2015 for a review). Other interventions aim to improve psychosocial outcomes of children with substance misusing parents through Behavioural Couples Therapy with parents, sometimes with a parent‐training component (e.g., Kelley & Fals‐Stewart, 2002). Other interventions in this focused category include pharmacological treatment of the parent's substance misuse (e.g., Coyle et al., 2012; Fischer et al., 1999), school‐based psychoeducational programmes (e.g., Dore, Nelson‐Zlupko, & Kaufmann, 1999; Gance‐Cleveland & Mays, 2008) and attachment‐based parenting programmes (e.g., Luthar, Suchman, & Altomare, 2007; Suchman, DeCoste, McMahon, Rounsaville, & Mayes, 2011). In many instances, these interventions are delivered alongside and compared to standard treatment (e.g., methadone maintenance or usual case‐management practices).

### 1.3 How the intervention might work

Due to the wide range of interventions that will be included in this review, there are a number of possible mechanisms by which intervention might work. In a general sense, interventions for substance misusing parents are likely to impact child outcomes by modifying or reducing the impact of known risk factors, including parental psychopathology, parenting knowledge and skills, enhancement of the quality of the parent‐child relationship, involvement in the criminal justice or child welfare systems or impoverished environments. Different categories of interventions may impact child outcomes through more specific pathways. For example, interventions based on family disease models generally use a 12‐step model, focusing on abstinence, psychoeducation and knowledge as key mechanisms through which change can occur (for a review, see Usher, McShane, & Dwyer, 2015). In comparison, family prevention models target risk and protective factors linked with parent substance misuse to generate change (for a review, see Usher et al., 2015). Pharmacological interventions reduce ingestion of drugs, thereby reducing foetal exposure and improving birth and developmental outcomes (Minozzi, Amato, & Vecchi, 2013). Legal interventions, such as Family Treatment Drug Courts, aim to reduce the risks associated with parental substance misuse through providing support, whilst motivating behaviour change through incentives and penalties (Fay & Eggins, 2019). Therapeutic or psychosocial interventions also aim to improve child outcomes by reducing the risks associated with parental substance misuse, yet the theoretical models that underpin interventions in this category vary depending on the focus of the intervention (for reviews, see Dawe & Harnett, 2013; Neger & Prinz, 2015).

### 1.4 Why it is important to do the review

The current evaluation and review literature lacks integration and synthesis of the *relative* or comparative effectiveness of different intervention models that aim to improve the outcomes for children with substance misusing parents. Yet without a comprehensive and integrated synthesis of the extant evaluation literature, it will be difficult for practitioners, policy‐makers and researchers to focus their resources and decision‐making to improve the lives of children and families with substance misusing parents. As of December 2018, there were 24 existing reviews that (a) focus on interventions specifically for substance misusing parents and (b) have captured one or more studies that have assessed the impact of an intervention on child psychosocial outcomes. Although not all of these reviews adhere to a full systematic review methodology, each employs at least two systematic review techniques (e.g., systematic search, specific inclusion criteria and qualitative or quantitative synthesis of studies) and can be considered less biased than narrative reviews in the area (e.g., see, Choi, 2012; Marsh et al., 2011; Oliveros & Kaufman, 2011; Renk et al., 2015). These reviews highlight the range of interventions and large number of studies that are necessary for conducting an NMA. Existing reviews differ according to the specific intervention under consideration and whether only child outcomes or multiple different types of outcomes are included, and can be summarised as follows.

- One review examines the impact of home‐visiting interventions during pregnancy and the postnatal period for women with substance misuse issues and their impact across a range of parental and child outcomes (Turnbull & Osborn, 2012).
- Three reviews focus on Family Treatment Drug Courts for substance misusing parents with and their impact on child out‐of‐home placement (Llyod, 2015), child welfare system outcomes (Zhang, Huang, Wu, Li, & Liu, 2019) or child maltreatment outcomes (Eldred & Gifford, 2016).
- One review examines the impact of multidimensional interventions for substance misusing mothers and their impact on multiple child outcomes (Niccols et al., 2012).
- Four reviews focus on parenting interventions for substance misusing parents across multiple parent and child outcomes (Bowie, 2005; Moreland & McRae‐Clark, 2018; Neger & Prinz, 2015; Peisch et al., 2018).
- Two reviews and one Cochrane protocol concentrate on child‐focused preventative interventions for improving outcomes for children of substance misusing parents (Bröning et al., 2012) or alcohol misusing parents (Cuijpers, 2005; McLaughlin Aisling, Macdonald, Livingstone, & McCann, 2014).
- Two Cochrane reviews examine the impact of pharmacological interventions during pregnancy on maternal and child outcomes in the context of alcohol misuse (Smith, Lui, & Terplan, 2009) and opioid dependence (Minozzi et al., 2013).
- Several reviews capture a broad range of psychosocial interventions for parental substance misuse and their impact on multiple outcomes (including child outcomes) for either alcohol misuse during pregnancy (Lui, Terplan, & Smith, 2008; Scobie and Woodman, 2017; Stade et al., 2009) or all types of parental substance misuse (Austin & Osterling, 2006; Calhoun, Conner, Miller, & Messina, 2015; Heimdahl & Karlsson, 2016; McGovern, Addison, Newham, Hickman, & Kaner, 2017; Mitchell & Burgess, 2009; Murphy, Harper, Griffiths, & Joffrion, 2017; Templeton, Velleman, & Russell, 2010; Usher et al., 2015).

Although the existing review literature is extensive, there is variation in the degree of methodological quality and content coverage. We argue that issues pertaining to the methodologies used for existing reviews and gaps in content coverage reduce the ability to draw reliable conclusions about the effectiveness of interventions for improving psychosocial outcomes for children with substance misusing parents.

#### 1.4.1 Methodological limitations of existing reviews

Perhaps the most important methodological limitation of existing reviews is the lack of quantitative syntheses. Very few of the reviews with sufficient studies use meta‐analysis to synthesise the evaluation evidence (e.g., Minozzi et al., 2013; Niccols et al., 2012; Turnbull & Osborn, 2012; Zhang et al., 2019), despite the availability of multiple studies suitable for meta‐analysis across many of the reviews. Rather, authors provide qualitative summaries of intervention effectiveness that are based on the raw differences, statistical significance or effect sizes of individual studies. Although qualitative summaries are useful for assessing the breadth and qualities of intervention research, this methodology is inadequate for providing a reliable and precise estimate of an intervention impact (Borenstein, Hedges, Julian, & Rothstein, 2009; Littell, Corcoran, & Pillai, 2008).

Methodological limitations of existing reviews also highlight the need for an updated and more comprehensive systematic search. First, existing reviews may not provide an accurate representation of the most up‐to‐date intervention evidence because between 5 and 10 years have passed since the searches were conducted for many of the reviews. Second, there may be potential biases in the existing reviews. Some authors excluded studies that found negative intervention effects or only reported study outcomes if they were statistically significant. Others have introduced publication bias by either explicitly excluding documents not published in peer‐reviewed journals, omitting searches for unpublished literature or limiting searches to very few sources. In addition, some authors implemented restrictive searches, such as very limited search terms or multiple Boolean AND clauses. Third, much of the current body of reviews lacks transparency in the reporting of searches and sensitive search strategies. Many authors do not explicitly report their exact search and how it was implemented during their systematic search (e.g., what search fields were used).

#### 1.4.2 Content gaps in existing reviews

The current corpus of reviews does not provide complete coverage of the extant literature. Some reviews explicitly omit studies that include substance misusing fathers (e.g., Niccols et al., 2012), focus only on the prenatal period (e.g., Minozzi et al., 2013; Smith et al., 2009) or omit studies that contain child outcomes in the absence of parent‐level outcomes (e.g., Neger & Prinz, 2015). Others focus on alcohol misuse and do not capture equivalent interventions for populations with illicit drug misuse issues (e.g., Cuijpers, 2005; Lui et al., 2008; Smith et al., 2009; Stade et al., 2009; Templeton et al., 2010).

However, the most important limitation is that the existing review literature does not permit valid conclusions to be made about the *comparative* impact of these interventions for children with substance misusing parents. Yet understanding the relative impact of different interventions for a particular population is crucial for informing the decision‐making of both practitioners and policy‐makers (Hutton et al., 2015; Mavridis et al., 2015; Salanti, 2012). A recent methodological development, called NMA, provides an avenue for addressing these this important question. NMA, also known as multiple treatments meta‐analysis, has been referred to as “the next generation evidence synthesis tool” (Salanti, 2012, p. 80) and extends traditional pairwise meta‐analytic techniques. NMA provides an approach for (a) quantitatively synthesising both direct and indirect effects of multiple interventions for a particular population or condition; and (b) ranking interventions according to their effectiveness, even in the absence of trials that have directly compared the treatments (Mavridis et al., 2015; Salanti, 2012).

Our review will begin to address the above‐mentioned methodological quality and content coverage issues. Specifically, our review will both (a) enhance and update the existing body of reviews and (b) synthesise the *comparative* impact of interventions on the psychosocial outcomes of children with substance misusing parents. Provided sufficient data are available and the underlying analytical assumptions are satisfied, the proposed review will provide the first NMA that synthesises the relative impact of multiple interventions on the psychosocial outcomes for children with substance misusing parents. Importantly, the review will enable policy makers and practitioners to make informed and reliable choices between intervention models.

## 2 OBJECTIVES

The overarching objective of this review is twofold. First, we aim to enhance and update existing reviews by comprehensively synthesising the full array of psychosocial, pharmacological and legal interventions that aim to improve the psychosocial outcomes of children with substance misusing parents. Second, we aim to use NMA to integrate and examine the comparative impact of these interventions. Specifically, the review will address the following research questions.

- What is the comparative impact of psychosocial, pharmacological and legal interventions for improving the psychosocial outcomes of children with substance misusing parents?
- Does the impact of interventions vary according to the child developmental period (e.g., infancy, early childhood, adolescence) or the type of (a) outcome measure; (b) substance misuse; (c) practitioner implementing the intervention; or (d) intervention setting?
- Does the impact of interventions vary by the country of implementation?

## 3 METHODOLOGY

### 3.1 Criteria for including and excluding studies

#### 3.1.1 Types of study designs

Studies will be included in the review if they report on a quantitative impact evaluation of an eligible intervention using eligible participants and outcome measures. The impact evaluation must also utilise a randomised experimental design or methodologically robust quasi‐experimental design with an eligible comparison condition. Eligible comparison conditions are placebo, no treatment, waitlist control, treatment‐as‐usual and alternative treatment.

Key research synthesists advise against using traditional research design labels when delineating an inclusion threshold for nonrandomised studies in a systematic review (e.g., Higgins et al., 2012; Reeves, Deeks, Higgins, & Wells, 2011). Rather, the suggestion is that inclusion thresholds should be based on the design features of studies due to (a) the variation and possible ambiguity across disciplines in relation to research design terminology; and (b) the likelihood that risk of bias will affect specific design features versus an overall research design category. For the purposes of this review, methodologically robust quasi‐experimental designs are defined as those which permit causal inference by minimising threats to internal validity. For example, maximising treatment and comparison group equivalence through matching (e.g., propensity score matching), measurement of outcomes multiple times pre‐ and postintervention to reduce maturation threats (e.g., interrupted time‐series, cohort panel designs) or adjusting for confounding factors through statistical modelling (e.g., multiple regression, propensity score modelling). Due to serious threats to internal validity, single group studies with one preintervention and one postintervention outcome measure will be excluded from the review.

To be included in the meta‐analyses, study authors must report sufficient data to calculate an effect size. Where data are not reported, the required data will be sought by contacting the document authors. Should the data not be provided by study authors, the study will be excluded from meta‐analyses, but will be coded and included in study summary tables.

#### 3.1.2 Types of participants

This review will focus on families with children under the age of 18 who have one or more currently substance misusing parents. The primary research participants used in eligible impact evaluations must be either substance misusing parent(s), children of substance misusing parents or entire families characterised by parental substance misuse issues. For the purposes of this review, a parent is defined as an individual who is responsible for providing physical, emotional and/or financial care for a child. Teenage, biological, foster, adoptive or kinship caregivers are eligible for inclusion. A child is defined as an individual between the ages of 0–18 years who is under the care of at least one a parent, and a family is defined as at least one child and one parent.

Parents will be classified as “currently substance misusing” if they have been classified as such via standardised diagnostic criteria (e.g., DSM, ICD 10) or a self‐report measure (e.g., AUDIT). In the absence of classification supported by diagnostic or self‐report measures, studies will be included if the authors explicitly identify the research population as substance misusing parents. For example, a study would be included if the authors note that all study participants are methadone maintained mothers, even if the authors do not report formal diagnoses or baseline levels of substance use. Parents will be classified as substance misusing if they are misusing alcohol, illicit drugs and/or prescription drugs. If the study sample is not comprised completely of substance misusing parents, we will follow Turnbull and Osborn's (2012) approach, whereby the study sample must include at least 50% substance misusing parents to be included in the review.

#### 3.1.3 Types of interventions

In order to conduct an NMA, this review will include all possible psychosocial, pharmacological or legal interventions that explicitly aim to improve the psychosocial well‐being of families characterised by parental substance misuse. However, the focus of the review will be studies that examine the impact of interventions on child psychosocial outcomes. Examples of eligible interventions are described in the Section 1.2 (not exhaustive) and decisions regarding the categorisation of interventions will be based on the TIDieR Checklist (Hoffmann et al., 2014) and the specific intervention components coded for each eligible study (see Appendix). The categorisation approach will be reported in the final review and will guide assessment of the transivity assumption (Hutton et al., 2015). Based on existing literature in the area, we anticipate that the majority of the included studies will utilise a treatment‐as‐usual comparison condition (e.g., methadone maintenance, case‐management without the intervention under consideration). Interventions will be included irrespective of whether it is initiated during the prenatal or postnatal period. In addition, studies will be included if the intervention focuses on the misuse of alcohol, illicit drugs and/or prescription drugs.

#### 3.1.4 Types of outcome measures

In order to comprehensively synthesise the impact of eligible interventions on children with substance misusing parents, this review will include a broad range of outcomes nested under the banner of “psychosocial wellbeing”. Outcomes will be considered eligible if they are measured using standardised or nonstandardised instruments or consist of official, diagnostic, observation or self‐report data. Examples of primary outcomes include, but are not limited to, the following.

- Child development (e.g., attachment, language, cognitive functioning, educational outcomes).
- Child psychopathology (e.g., externalising/internalising behaviour, mental health diagnoses).
- Child maltreatment, abuse or neglect.
- Child antisocial behaviour (e.g., truancy, delinquency, illicit drug use).
- Other child psychosocial well‐being outcomes (e.g., self‐esteem).

The decision to utilise one intervention over another may rest on other considerations beyond the effectiveness of the intervention, such as intervention cost, resource intensity or degree to which participants accept or complete treatment. Therefore, if reported in eligible studies, the following secondary outcomes will also be coded and analysed: cost‐effectiveness, treatment completion, length of time in treatment and acceptability of treatment (e.g., participant perspectives of the intervention).

#### 3.1.5 Duration of follow‐up

Studies will be included irrespective of the length of follow‐up after the intervention. Where the length of follow‐up varies across studies, we will group and synthesise studies according to similar follow‐up durations. For example, short (e.g., 0–3 months postintervention), medium (>3 months, <6 months) and long‐term follow‐up (>6 months postintervention).

#### 3.1.6 Types of settings

There will be no restrictions on the intervention setting or treatment format (e.g., inpatient, outpatient, community settings, family home, online or computerised, one‐on‐one or group settings). The review will include intervention studies conducted in any geographical location or country and published in any language, provided translation can be undertaken to code eligible studies.

#### 3.1.7 Additional eligibility criteria

Studies that satisfy the above‐mentioned eligibility criteria will be included in the review regardless of publication status. While documents written in languages other than English will not be excluded from the review, they will only be assessed for final eligibility and included in the syntheses if a translation can be sourced.

### 3.2 Search strategy

During a piloting phase, the search mentioned below provided the optimum balance between sensitivity and specificity. Wherever possible, the search terms within the columns in Table 1 will be combined with Boolean OR and enclosed in brackets. The grouped terms will then combined with Boolean AND. The search string will be applied to the title, abstract, keyword and indexing term/subject heading search fields. For example: ((**TI:**(alcohol* OR *amphetamine*….) OR **AB:**(alcohol* OR *amphetamine*….) OR **KW:**(alcohol* OR *amphetamine*….) OR **Index:**(alcohol* OR *amphetamine*….)) AND (**TI:**(“care*giver*” OR caregiver*….) OR **AB:**(“care*giver*” OR caregiver*….) OR **KW:**(“care*giver*” OR caregiver*….) OR **Index:**(“care*giver*” OR caregiver*….)) AND (**TI:**(experi* OR randomi*….) OR **AB:**(experi* OR randomi*….) OR **KW:**(experi* OR randomi*….) OR **Index:**(experi* OR randomi*….))).

**Table 1:** Systematic search terms

Substance terms		Population terms	Impact evaluation terms
addict*	marij*	“care NEAR/2 giver*”	compar*
acid*	MDMA	families	crossover
alcohol*	meth	family	effecti*
*amphet*	methadone	father*	efficac*
benzo*	narco*	maternal	evaluat*
buprenorphine	opiate*	mother*	group*
cannab*	opioid*	parent*	*experiment*
cocaine	oxy*	paternal	interven*
crack	overdos*		match*
drink*	pharma*		meta*
drug*	polydrug*		pair*
GHB	polysubstance*		pilot*
ecstasy	prescri*		program*
fentanyl	pseudo*		“propensity score*”
hallucino*	psychoactive		*random*
heroin	speed*		RCT
inhalant*	stimulant*		review*
illict*	*substance*		service*
inject*	THC		therap*
intoxica*	tranquil*		train*
ketamine	weed		treat*
LSD			trial*

Due to differences in the search operators and indexing systems across electronic databases, the exact search will be customised to each location and may slightly vary across search locations. Where the functionality of a search location does not permit complex search strategies, a simplified version of the search will be utilised. The search will place no limits on publication date, document language or publication status. However, clearly ineligible document types will be excluded from search results if the specific search location permits (e.g., book reviews). Each search will be recorded in a search record as per recommended guidelines (see Kugley et al., 2017; Littell et al., 2008).

To reduce disciplinary and publication bias, the systematic search will cover multiple disciplines and search sources (see Table 2 for search locations). The following additional search strategies will also be used to identify eligible documents not already captured.

1. Reference harvesting of all eligible studies and previous reviews.
2. Forward citation search/citation tracking for all eligible studies.
3. Contacting prominent scholars relevant to the review topic to enquire about eligible documents not yet published or disseminated.
4. Hand‐searching the two most recent issues of key journals to identify potentially eligible documents not yet indexed in academic databases (see Table 2).

**Table 2:** Systematic search locations

Academic databases	Campbell Collaboration Library of Systematic Reviews
	Cochrane Collaboration Library of Systematic Reviews
	Database of Abstracts of Reviews of Effectiveness (DARE)
	HeinOnline (Law Journal Library)
	ScienceDirect
	Scopus
	EBSCO platform
	Criminal Justice Abstracts
	Medline
	Web of Science platform:
	Conference Proceedings Index
	Current Contents Connect
	Medline
	Social Science Citation Index
	ProQuest platform
	Criminal Justice Database
	Dissertation and Theses Global
	Family Health
	Health and Medical Complete
	International Bibliography of the Social Sciences
	Nursing and Allied Health
	Psychology Journals
	Research Library
	Social Science Database
	Sociological Abstracts
	Social Services Abstracts
	OVID platform
	Cumulative Index to Nursing and Allied Health Literature (CINAHL)
	Embase
	Joanna Briggs Institute EBP Database
	PsycINFO
	PsycEXTRA (grey literature)
	Informit platform
	Australian Criminology Database (CINCH)
	DRUG
	Family and Society Abstracts (FAMILY)
	Health and Society
	Health Collections
	Humanities and Social Sciences Collection
Grey literature sources and websites	Action on Addiction
	Alcohol and Alcohol Science Database (ETOH)
	Alcohol Concern (UK)
	Alcohol Research (UK)
	American Institutes for Research
	AODstats.org.au
	Australian Centre for Child Protection
	Australian Institute of Family Studies
	Australian Therapeutic Jurisprudence Clearinghouse
	Bibliomap
	California Evidence‐Based Clearinghouse for Child Welfare
	Canadian Research Institute for Social Policy (CRISP)
	CareData
	CEBC (California Evidence‐Based Clearinghouse for Child Welfare)
	CEBI (Centre for Evidence‐Based Intervention, Oxford University)
	Centre for Evidence‐based Public Health Policy
	Child Abuse and Neglect Digital Library (canDL)
	Child Abuse, Child Welfare & Adoption Database
	Child Trends
	Child Welfare Information Gateway
	ChildData
	DART‐Europe E‐theses Portal
	Database of Promoting Health Effectiveness Research (DoPHER)
	Drugscope
	e‐Theses Online Service (eThOS)
	Early Intervention Foundation (www.eif.org.uk)
	Economic and Social Research Council (ESRC, Regard database)
	European Monitoring Centre for Drugs and Drug Addiction (EMCDDA)
	Evidence for Policy and Practice Information & Coordinating Centre (EPPI‐Centre)
	Family Drug Support Australia (www.fda.org.au)
	FLoSse Research
	Foundation for Alcohol Research and Education (Australia)
	Grey Literature Network Service
	Health Technology Assessment Database (HTA)
	Intute: Social Science
	Institute of Alcohol Studies
	Joseph Rowntree Foundation
	MDRC (https://www.mdrc.org/publications)
	National Centre on Substance Abuse and Child Welfare
	National Child Traumatic Stress Network
	National Criminal Justice Reference Service (NCJRS)
	National Drug and Alcohol Research Centre (NDARC, Australia)
	National Institute on Alcohol Abuse and Alcoholism
	National Institute on Drug Abuse (NIDA, US)
	National Institute for Health and Care Excellent (NICE)
	NPC Research (http://npcresearch.com/specialty-areas/)
	National Research Register (NRR, National Health Service, UK)
	National Society for the Prevention of Cruelty against Children (NSPCC)
	National Technical Information Service (NTIS)
	Networked Digital Library of Theses and Dissertations
	NHS Economic Evaluation Database (EED)
	OAIster
	OpenDOAR
	OpenGrey
	Parent Mental Health systematic map database (hosted by EPPI‐Centre)
	PubMed
	ProjectCork.org
	RAND Drug Policy Research Centre
	Register for Open Access Repositories (ROAR)
	SAMHSA's National Registry of Evidence‐Based Programs and Practices
	Save the Children
	Social Care Online
	Social Care Institute for Excellence (SCIE, including ELSC)
	Social Sciences Literature Information System (SOLIS)
	Social Science Research Network (SSRN)
	The Evidence Network
	The Urban Institute
	Turning Research into Practice (TRIP database)
	Turning Point
	What Works Clearinghouse
	What Works for Children
	United Nations Office on Drugs and Crime (UNODC)
Trial registries	Australian and New Zealand Clinical Trials Registry
	ClinicalTrials.gov
	Clinical Trials Results
	Cochrane Central Register of Controlled Trials (CENTRAL)
	ISRCTN Registry (controlled-trials.com)
	NIH RePORTER
	Trials Register of Promoting Health Interventions (TRoPHI)
	Unreported Trials Register
	UK Clinical Research Network (UKCRN Study Portfolio)
	WHO International Clinical Trials Registry Platform
Hand searched journals	Addiction
	Child Abuse and Neglect
	Child Abuse Review
	Child and Adolescent Social Work Journal
	Child Maltreatment
	Children and Youth Services Review
	Journal of Drug Issues
	Journal of Experimental Criminology
	Substance Abuse

### 3.3 Description of methods used in primary research

Based on the existing corpus of reviews, we anticipate that included studies will employ either randomised or nonrandomised experimental designs with a range of comparison conditions (e.g., treatment‐as‐usual, alternative treatment, waitlist control). In addition, we anticipate identifying studies that assess intervention impact using a broad range of child psychosocial outcomes measured in a continuous and/or categorical manner.

### 3.4 Criteria for determination of independent findings

There are three areas of consideration for determining independence of study findings: multiple reports of the same study, multiple outcomes for the one study and research designs with clustering. The software that will be used to manage this review enables nesting of multiple dependent documents pertaining to one study. If dependent studies are identified, all studies will be coded and data will be extracted from the most complete report of the study and the study will only be included once in each meta‐analysis for each conceptually unique outcome category. Where studies report on multiple conceptually similar outcomes, the effect sizes will be combined into a single effect size using the method described by Borenstein et al. (2009). This composite effect size will then be used in the meta‐analysis. If a study utilises a research designs with clustering (e.g., study sites assigned to conditions), the method suggested by Fu et al. (2013) and Higgins et al. (2011) will be used to adjust the standard error. If the included study does not report the required intraclass correlation coefficient (ICC), we will use the approach taken by Barlow, Bergman, Kornør, Wei, & Bennett (2016) to assess the impact of clustering on effect estimates. Specifically, Barlow et al.'s (2016) systematic review of group‐based parenting interventions took the approach of conducting sensitivity analyses to examine whether the results of their meta‐analyses varied with ICCs of 0, .03, .02 and .1.

### 3.5 Details of study coding categories

#### 3.5.1 Title and abstract screening

The first phase of assessing study eligibility will be comprised of screening the titles and abstracts identified in the systematic search. After removing duplicates and clearly ineligible document types (e.g., book reviews), records captured by the systematic search will be imported into the *SysReview* review management software (Higginson & Neville, 2014). Each title and abstract (record) will then be assessed according to the following exclusion criteria.

1. Ineligible document type (e.g., book review).
2. Document is not unique.
3. Document is not about parental substance misuse.

Although all efforts will be made to remove ineligible document types and duplicates prior to screening, automated and manual cleaning can be less than perfect. Therefore, the first two exclusion criteria will be used to remove ineligible document types and duplicates prior to screening each record on substantive content relevance.

The full‐text document for each record retained at the title and abstract screening stage will then be attached within *SysReview* before progressing to full‐text eligibility screening. If full‐text documents cannot be retrieved via existing university resources, they will be ordered through the university libraries of the review authors or by contacting study authors.

#### 3.5.2 Full‐text eligibility screening

Each full‐text document progressed from the title and abstract screening stage will be screened for final eligibility according to the following exclusion criteria.

1. Ineligible document type (e.g., book review).
2. Document is not unique.
3. Ineligible participants.
4. Ineligible outcome measure(s).
5. Not a quantitative impact evaluation of an eligible intervention using eligible participants or outcomes.
6. Ineligible research design.

Although all efforts will be made to remove ineligible document types and duplicates in prior stages, these types of records can sometimes progress into later stages, for example, where duplicate records are not adjacent to each other during screening or where screeners cannot unequivocally determine if record is ineligible based on the title and abstract. Therefore, the first two exclusion criteria will be used to remove ineligible document types and duplicates prior to screening each document for final eligibility.

#### 3.5.3 Full‐text coding and risk of bias assessment

Eligible studies progressing from the full‐text screening stage will be independently double‐coded using the coding protocol provided in the Appendix. Broadly, studies will be coded according to the following domains.

1. General study characteristics (e.g., document type, study location).
2. Participants (e.g., sample characteristics by condition).
3. Intervention (e.g., intervention components, intensity, setting).
4. Outcomes (e.g., conceptualisation, mode of measurement, time‐points).
5. Research methodology (e.g., design, unit and type of assignment).
6. Effect size data.
7. Risk of bias.

Risk of bias will be assessed using either the Cochrane randomised or nonrandomised risk of bias tools (Higgins et al., 2011; Sterne et al., 2016), whereby studies will be rated across domains as having high, low or unclear risk of bias. If a domain is rated as “unclear”, study authors will be contacted to obtain missing data. Results of the risk of bias assessment will be presented in summary tables and in a risk of bias summary figure. Depending on the data available, sensitivity analysis will be used to examine the impact of risk of bias on effect estimates and corresponding confidence intervals. Potential analyses include: forest plots stratified by level of risk, moderator analysis or metaregression. The approach taken to incorporate risk of bias in statistical analyses will be depend on the degree of variation in risk of bias across included studies. For example, statistical analysis may be stratified by level of risk or all studies may be included in one analysis with a narrative discussion of the risk of bias (see Higgins et al., 2011 for more detail).

### 3.6 Statistical procedures and conventions

Statistical analyses will be performed in R using the *netmeta* program code available at http://cran.r-project.org/web/packages/netmeta/netmeta.pdf (Rücker, Schwarzer, Krahn, König, & Schwarzer, 2015). We anticipate that primary studies will report a variety of data for calculation of effect sizes. Where outcomes are reported as continuous measures, Hedges *g* (standardised mean differences [SMDs]) will be computed, along with 95% confidence intervals. For studies with binary outcomes, effect sizes will be computed as odds ratios and then transformed into SMDs for meta‐analyses (see Borenstein, Hedges, Higgins, & Rothstein, 2009). For all other studies not falling into these categories, we will use David B. Wilson's effect size calculator to calculate SMDs for meta‐analyses.

We will follow the recommendations of the Campbell Collaboration Methods Group and PRISMA Group when conducting and reporting on networked statistical analyses (Hutton et al., 2015; Wilson, Tanner‐Smith, & Mavridis, 2016). Specifically, the geometry of the network(s) will be presented in network diagrams, whereby nodes represent competing interventions and/or comparisons and the lines connecting the nodes reflect direct comparisons between nodes (Chaimani, Higgins, Mavridis, Spyridonos, & Salanti, 2013; Mavridis et al., 2015). The weight of the lines in network diagrams will represent the number of studies with the respective comparison and the size of the nodes will reflect the number of studies involving the respective intervention. The transivity assumption will be assessed by examining the consistency between the direct and indirect evidence, drawing on a global test approach (see Hutton et al., 2015 for a review) and inconsistency plots (see Chaimani et al., 2013; Mavridis et al., 2015). The relative effects of each pair of interventions will be summarised in league tables, and intervention rankings will be displayed using rankograms and ranking plots, with accompanying surface under the cumulative ranking curve values (see Hutton et al., 2015; Mavridis et al., 2015). Where there is inconsistency detected, intervention rankings will be interpreted with caution.

It is important to note that NMA assumes that there are effect sizes that compare (a) eligible treatments and comparison groups; and (b) eligible treatments with other eligible treatments. If there is an absence of the latter, it may be more appropriate to conduct moderator analyses used in standard meta‐analyses, which are conducted using either the analogue to the analysis of variance (ANOVA) or metaregression approach. Both approaches require sufficient studies to reach statistical power, but the metaregression approach will be the preferred approach due to the ability use multiple variables which may impact effect size estimates (e.g., design features, risk of bias, focus of treatment).

Due to the difficulties that can be associated with meeting the transivity assumption, consistency assumption and types of within study comparisons, it is important to specify an alternative a priori analysis approach. One part of the problem stems from only a preliminary understanding the extant evaluation literature that may be eligible for the review when finalising a review protocol. For example, the systematic review may identify nuances in the interventions and outcome categories that require an approach that was not anticipated at the protocol stage. We anticipate conducting separate network meta‐analyses for each conceptually distinct outcome category that will combine all comparison conditions. However, should this not be possible, we will conduct traditional meta‐analyses that examine each broad intervention model as compared to (a) eligible comparison conditions; and (b) alternative treatments. To categorise interventions, we will use the TIDieR Checklist (Hoffmann et al., 2014), along with the intervention components using the coding guide in the Appendix, to ensure that intervention categories are internally consistent.

For studies reporting multiple points of follow‐up, effect sizes will be calculated for each time‐point, but synthesised separately with studies that have similar outcome time‐points. If included studies report baseline and postintervention outcome data, SMDs will be calculated using baseline adjusted mean differences (i.e., mean change scores) and the change score standard deviations, will be standardised using the raw standard deviation within groups. Where authors do not report the standard deviation for mean change scores, Lipsey and Wilson's (2001) formula will be used to calculate the standard deviation $\left(\frac{s_{T1+}^{2}s_{T2}^{2}/2}{\sqrt{2(1-r)}}\right)$. If studies report follow‐up outcome data, postonly outcome data will be used to estimate SMDs, and follow‐up outcomes will be analysed separately from postintervention outcomes.

Heterogeneity between studies will be examined using the approach suggested by Chaimani et al. (2013). Specifically, τ^2^ will be used to express heterogeneity across the network, and we will present the NMA mean summary effects in forest plots with corresponding confidence and predictive intervals to assess whether the magnitude of heterogeneity impacts the mean summary effects. Moderator analyses will be used to explore potential sources of heterogeneity. Specifically, the analogue to ANOVA will be used for categorical moderators and regression‐based approaches will be used for continuous moderators. Depending on the data reported in included studies, additional exploratory subgroup analyses may be performed; however, we will clearly distinguish between a priori and exploratory analyses in our reporting. The specific a prior moderator variables are: child developmental period (e.g., infancy, early childhood, adolescence); type of outcome measure (e.g., mental health, child welfare placement, child abuse or neglect); type of parental substance misuse (e.g., alcohol, drug, polysubstance); type of practitioner implementing the intervention; type of intervention setting (e.g., home, school, community, residential facility); and country of implementation.

Assessment of publication bias will be the final stage of analysis and will first entail inspection of comparison‐adjusted funnel plots for asymmetry to identify whether effect size estimates are influenced by publication bias (see Chaimani et al., 2013; Mavridis et al., 2015). If asymmetry is detected, subgroup analyses will be conducted to assess whether effect sizes significantly differ by publication status of the included studies.

## SOURCE OF SUPPORT

This review is partially supported by the Australian Research Training Program Domestic Fee Offset awarded to Elizabeth Eggins for March 2015–August 2018.

## CONFLICT OF INTERESTS

Sharon Dawe has been involved in the development and evaluation of the Parents Under Pressure programme (PuP). PuP has been evaluated in families with parental substance misuse issues and would meet the inclusion criteria for the review. She has also been an author on commissioned monographs focusing on the impact of parental substance misuse on child outcomes and on the impact of substance misuse on other family members. To minimise any potential biases, other review authors will screen and coded any eligible studies coauthored by Sharon Dawe.

## AUTHOR CONTRIBUTIONS

All the authors developed the content and contributed to systematic review methods. E. E. and D. B. W. provided statistical analysis. E. E., N. C.‐M., and J. B. contributed to information retrieval. Collectively, the authors of the proposed review provide a distinctive combination of policy, practice, programme evaluation and systematic review expertise. Elizabeth Eggins will be responsible for initiating and overseeing updates of the review. She is the Managing and Associate Editor for the Campbell Collaboration Crime and Justice Coordinating Group and has coauthored and managed a range of research projects grounded in systematic review methodology, including Campbell Collaboration and industry‐funded systematic reviews. Her PhD research focuses on building the evidence‐base for interventions implemented with families characterised by parental substance misuse. Sharon Dawe is a Professor of Clinical Psychology with substantial expertise in addiction science. She is a coauthor for several systematic reviews in the area of vulnerable families. She has made, and continues to make, significant contributions across research, policy and practice arenas (e.g., by serving on expert panels, training practitioners and publishing addiction science research). David Wilson an expert in research synthesis and Methods Editor for the Campbell Collaboration Crime and Justice Co‐Ordinating Group (previous Co‐Chair). He has made significant contributions in relation to systematic review methodology, programme evaluation and systematic reviews in the area of crime and justice (including substance misuse). Ned Chandler‐Mather and Jospeh Betts are coauthors of other Campbell Collaboration reviews and have experience in implementing systematic review protocols.

## APPENDIX 1 FULL‐TEXT CODING FORM ^1^ This form has been informed by published coding forms (e.g., Littell et al., 2008; Fay & Eggins (2019); Betts et al. (2019); Mitchell, Wilson, Eggers, & MacKenzie, 2012).

### General study details

1. Study ID [*textbox*]
2. Report ID [*textbox*]*
3. What type of document is this study? [*dropdown menu*]
  a. Peer‐reviewed journal article
  b. Book chapter
  c. Dissertation
  d. Conference presentation
  e. Government report, technical report or working paper
  f. Other (specify in textbox)
4. How was this study located during the search process? [*dropdown menu*]

a. Systematic search of electronic database
b. Systematic search of nonacademic database
c. Hand‐search or reference harvesting
d. Professional contact
e. Other (specify in textbox)

5. In what country was the intervention implemented? [*textbox*]
6. How many courts were included in the study? [*textbox*]
7. If the evaluation and/or intervention was funded, record the funding source. [*textbox*]

*SysReview allows for multiple reports of a single study to be included in the one full‐text coding record form. Each report is nested within the overall study record and the Report ID will consist of the Study ID followed by a unique alphabetical code (e.g., 1234_a, 1234_b…).

### Participants

1. Who are the participants? [*checkboxes*]
  a. Parents (mothers only)
  b. Parents (fathers only)
  c. Parents (both mothers and fathers)
  d. Other caregiver (e.g., foster parents, grandparents)
  e. Children
  f. Other (specify in textbox)

2. If applicable, describe the recruitment and sample for parent(s)/caregiver(s) using the fields below:
  a. How were participants recruited? [*textbox*]
  b. What were the eligibility criteria for inclusion in the study? [*textbox*]
  c. Describe the sample attrition. [*textboxes*]
    **Table:**
    **Number of participants**	**Treatment**	**Comparison**	**Total**
    Referred to study			
    Consented			
    Assigned			
    Began intervention			
    Completed intervention			
    Completed Follow‐up 1			
    Completed Follow‐up 2 (if applicable)			
  d. Describe the characteristics of the sample. [*textboxes*]
    **Table:**
    **Characteristic**	**Treatment**	**Comparison**	**Total**
    Age (mean, *SD*, range)			
    Gender (% female)			
    Ethnicity (proportions)			
    Socioeconomic status			
    Comorbidity			
  e. Note any other pertinent sample information (e.g., parity, marital status, education, prior criminal history, type of drug use, addiction history or other key risk factors present). Please record for both the treatment and comparison groups [*textbox*]

3. If applicable, describe the recruitment and sample for children using the fields below:
  a. How were participants recruited? [*textbox*]
  b. What were the eligibility criteria for inclusion in the study? [*textbox*]
  c. Describe the sample attrition. [*textboxes*]
    **Table:**
    **Number of participants**	**Treatment**	**Comparison**	**Total**
    Referred to study			
    Consented			
    Assigned			
    Began intervention			
    Completed intervention			
    Completed Follow‐up 1			
    Completed Follow‐up 2 (if applicable)			
  d. Describe the characteristics of the sample. [*textboxes*]
    **Table:**
    **Characteristic**	**Treatment**	**Comparison**	**Total**
    Age (mean, *SD*, range)			
    Gender (% female)			
    Ethnicity (proportions)			
    Comorbidity			
  e. Note any other pertinent sample information (e.g., placement status, other key risk factors present). [*textbox*]

4. How was parental substance misuse substantiated? [*textbox*]
5. What type of substance misuse was captured by the study? [*dropdown menu*]
  a. Alcohol
  b. Drug (specify in textbox)
  c. Both

### General methodological details and nature of comparisons

1. What is the nature of the comparisons for this study?
  a. Single intervention contrasted with single comparison condition
  b. Multiple interventions against a single comparison condition
  c. Within one group over time
  d. Other (specify in textbox)

2. General research design classification [*dropdown menu*]
  a. Randomised controlled trial
  b. Quasi‐randomised controlled trial
  c. Nonrandomised controlled trial (e.g., interrupted time‐series, matched control group design)
  d. Other (specify in textbox)

3. What type of comparison condition was used? [*dropdown menu*]
  a. No treatment
  b. Treatment‐as‐usual (specify in textbox)
  c. Alternative treatment (specify in textbox)
  d. Waitlist control
  e. Other (specify in textbox)

4. How were treatment and comparison groups formed? [*dropdown menu*]
  a. Random allocation
  b. Matching (specify matching method and matching variables in textbox)
  c. On basis of score on a specific measure (e.g., diagnosis, specify in textbox)
  d. Self‐selection
  e. Other (specify in textbox)
  f. Unclear

5. What was the unit of allocation? [*dropdown menu*]
  a. Participant
  b. Dyads
  c. Family
  d. Service site
  e. Other (specify in textbox)
  f. Unclear

6. If participants were randomly allocated to conditions, how was this implemented? [*dropdown menu*]
  a. Simple
  b. Yoked pairs
  c. Cluster (specify cluster in textbox)
  d. Block/stratified (specify variables in textbox)
  e. Matched pairs (specify matching variables in textbox)
  f. Other (specify in textbox)
  g. Unclear
  h. Not applicable

7. Who executed the randomisation? [*dropdown menu*]
  a. Researchers
  b. Practitioners
  c. Other (specify in textbox)
  d. Unclear

8. If applicable, was randomisation equivalent across intervention sites? [*dropdown menu*]
  a. Yes
  b. No
  c. Unclear
  d. Not applicable

9. Was group equivalence assessed?
  a. Yes (specify how this was done in textbox)
  b. No
  c. Unclear
  d. Not applicable

10. Were the treatment and comparison groups equivalent at baseline?
  a. Yes
  b. No (specify differences)
  c. Unsure
  d. Not applicable

11. Are there any differences between participants who completed versus did not complete the treatment?
  a. Yes (specify differences)
  b. No
  c. Unsure
  d. Not applicable

12. What was the unit of analysis? [*dropdown menu*]
  a. Participant
  b. Dyads
  c. Family
  d. Service site
  e. Other (specify in textbox)
  f. Unclear

### Intervention details

1. What is the name of the intervention(s), as reported by study authors? [*textbox*]
2. What settings were used during the intervention(s) (e.g., home, community, inpatient facility, school)? [*textbox*]
3. When was the intervention conducted (e.g., year)? [*textbox*]
4. Describe the intervention provided to participants. [*textbox*]
5. Describe the duration of the entire intervention. If available, describe the minimum, maximum, mean and standard deviation for intervention duration. [*textbox*]
6. Describe the intensity of the intervention (e.g., frequency of contacts and length of contacts). If available, describe the minimum, maximum, mean and standard deviation for intervention intensity. [*textbox*]
7. Who implemented the intervention? [*dropdown menu*]
  a. Nurse
  b. Social worker
  c. Psychologist
  d. Medical practitioner
  e. Other allied health practitioner
  f. Unclear
  g. Other (specify in textbox)

8. Was there more than one intervention site? [*dropdown menu*]
  a. Yes (specify number of sites in textbox)
  b. No
  c. Unclear

9. If possible, classify the intervention according to [dropdown menu]
  a. Family Disease Model (i.e., 12‐step, focus on abstinence and education)
  b. Family Prevention Model (i.e., focus on risk and protective factors in a holistic sense)
  c. Unclear

10. Was treatment integrity monitored? [*dropdown menu*]
  a. Yes (specify in textbox)
  b. No
  c. Unclear

11. Were there any issues with fidelity? [*dropdown menu*]
  a. Yes (specify in textbox)
  b. No
  c. Unclear

12. Did the authors report cost‐benefit data? [*dropdown menu*]
  a. Yes (specify in textbox)
  b. No
  c. Unclear

### Outcome(s) measurement*

*To be completed for each eligible outcome within a study (or group of reports for a study). To add another outcome, click the “Add another outcome” button located at the bottom of the screen.

1. What is the outcome being measured? [*textbox*]
2. What is the variable name that will be used in statistical software? [*textbox*]
3. Who does this outcome relate to? [*dropdown menu*]
  a. Parent/caregiver
  b. Child
  c. Other (specify)

4. How was the outcome measured (e.g., name of scale)? [*textbox*]
5. What are the psychometric properties of the measurement tool (e.g., reliability, validity, diagnostic thresholds, what higher/lower values mean)? [*textbox*]
6. How was the outcome data gathered? [*dropdown menu*]
  a. Self‐report
  b. Observation
  c. Official source (e.g., child protection status)
  d. Interview
  e. Other (specify in textbox)

7. Who was the respondent/participant? [*dropdown menu*]
  a. Child
  b. Parent/caregiver
  c. Teacher
  d. Practitioner
  e. Other (specify in textbox)

8. At what time‐point(s) was the outcome measured? [*textbox*]
9. Were data collected in the same manner for the treatment and comparison conditions? [*dropdown menu*]
  a. Yes
  b. No (specify in textbox)
  c. Unclear

10. Which condition does the raw difference/effect favour (ignore statistical significance)? [*dropdown menu*]
  a. Experimental condition
  b. Comparison condition
  c. Neither condition (no difference)
  d. Unclear

11. In which direction did the outcome change? [*dropdown menu*]
  a. Positive
  b. Negative
  c. Mixed (specify in textbox)
  d. Unclear

12. Were there statistically significant differences for this outcome? [*dropdown menu*]
  a. Yes
  b. No
  c. Not tested
  d. Unclear

13. What were the study author(s)’ conclusions about this outcome? [*textbox*]

### Effect size data*

*To be completed for each eligible outcome within a study (or group of reports for a study). To add another outcome, click the “Add another outcome” button located at the bottom of the screen.

1. On what page number is the effect size data reported? [*textbox*]
2. What type of effect size is being coded? [*dropdown menu*]
  a. Baseline or pre‐test measure prior to intervention)
  b. Posttest (first point of measurement after intervention)
  c. Follow‐up (subsequent point of measurement after first posttest)

3. What is the timeframe captured for the measure?
  a. Minimum [*textbox*]
  b. Maximum [*textbox*]
  c. Mean [*textbox*]
  d. Same for all participants (i.e., fixed) [*textbox*]

4. How was the effect size obtained for this outcome? [*dropdown menu*]
  a. Reported in document → *Go to Question 3*
  b. Calculated by user → *Go to Question 4*

5. Identify the type of effect size reported for this outcome and enter the required data for that effect size in the text boxes provided. [*textboxes*]
6. Enter the appropriate data in the relevant “Data for effect size calculations” tabs (see below). The data entered will depend on what is reported in the document. If none of the circumstances in the tabs reflect the data in the document, follow the link to David Wilson's online effect size calculator to calculate an effect size. You can enter the data in the “Data for effect size calculations 2” tab in the “Other information” textbox. [*textboxes*]
